# Acynetobacter baumanii extremely resistant outbreak control in ICU: a multidisciplinary approach

**DOI:** 10.1186/2197-425X-3-S1-A132

**Published:** 2015-10-01

**Authors:** M Muñoz, O Moreno, E Yuste, ME Poyatos, R Ramirez, S Narbona

**Affiliations:** Hospital Universitario San Cecilio, ICU, Granada, Spain

## Objective

Description of an Acynetobacter baumanii extremely resistant (A. Baumanii XDR) outbreak detection and management at the ICU of an University Hospital (Spain).

## Methods

Observational analysis using ENVIN-HELICS database of all the infected and/or colonized patients in our ICU between January and May 2014 and to describe the multi-disciplinary measures performed for it´s control: hygiene measures (strict hand hygiene, patients daily wash with special chlorhexidine gel, strict contact isolation), clustering measures (grouping all positive patients in ICU and hospitalization ward), general measures (high level structure cleaning, staff members training, adjusting nursing workload), optimize microbiological monitoring (fast culture and resistance maps processing), follow up during and after hospitalization, early empirical and directed antibiotic therapy.

## Results

The first two patients in whom infection/colonization by A. Baumanii XDR (strains OXA 51 and OXA 23) was detected, were hospitalized in vascular and general surgery units between November and December 2013. From January 2014 the sample increased until it was detected in 31 patients (45% colonized and 55% infected), 18 (58%) of which were admitted to ICU. APACHE II at admission was 23 ± 6.75, CI95(19.2-27.16). 91% of patients where hospitalized. 50% had risk factors at admission, like complicated abdominal surgery and/or pneumonia. 100% of ICU patients with positive cultures required mechanical ventilation (MV), central venous catheter and urinary catheterization. In this group, mortality rate was 33% (6 patients), 3 in the ICU and 3 afterwards in hospitalization ward. The fast detection and comprehensive set of measures allowed the ICU outbreak control in only four months, although in hospital required a longer time for total eradication.

## Conclusions

A fast detection and multidisciplinary team and measures application was essential to eradicate this ICU A. Baumanii XDR outbreak in only four months. 100% of patients had invasive instrumentation and MV.

The severity of A. Baumanii XDR infection is characterized by an increase in mortality, ICU and hospital length of stay.

